# Antibacterial Activity of Elephant Garlic and Its Effect against U2OS Human Osteosarcoma Cells

**Published:** 2013-10

**Authors:** Zehao Huang, Jianwu Ren

**Affiliations:** 1 Beijing Key Laboratory of Forest Food Processing and Safety, Beijing Forestry University, Beijing 100083, China; 2 Jain-Wu Ren, College of Biological Sciences and Biotechnology, Beijing Forestry University Beijing 10083, China

**Keywords:** Anticancer, Bacteriostasis, Elephant garlic, Osteosarcoma

## Abstract

***Objective(s):*** The present study was designed to investigate the antibacterial function and pharmacological effect of elephant garlic (*Allium ampeloprasum *var.* ampeloprasum*) on U2OS human osteosarcoma cells.

***Materials and Methods:*** Seven kinds of bacteria were reconstituted, inoculated and tested in this research to evaluate elephant garlic antibacterial activity. By the means of FACS analysis, cell proliferation assay, confocal laser scanning microscopy and Transwell migration assays, the effect of elephant garlic against U2OS human osteosarcoma cells was unveiled.

***Rerults:*** The antimicrobial activity of elephant garlic was stronger than ampicillin when used against *Escherichia coli, Bacillus subtilis, Bacillus thuringiensis, Staphylococcus actinomycetes, *and* gray actinomycetes.* Even at a very low concentration (12.5%), elephant garlic still had an antibacterial effect on common bacteria *E. coli* and *S. aureus*. The G0/G1 ratio of elephant garlic treated group cells increased while S phase decreased. Elephant garlic extract inhibited the growth of human osteosarcoma cells, U2OS, through preventing the transition from G1 phase to S phase. It reduced osteosarcoma cell, U2OS, invasion ability and significantly increased the proportion of apoptosis. It significantly affected the cytoskeleton generation.

***Conclusion: ***Elephant garlic exhibits antibacterial property and has an inhibitory effect on osteosarcoma cells (U2OS) proliferation and cell activity, suggesting the mechanism of its anticancer effects on U2OS human osteosarcoma cells.

## Introduction

Elephant garlic is a plant belonging to the *Allium* genus. It is actually a variant of the species to which the garden leek belongs (1). A bulb of elephant garlic weights up to a pound. Since its flavor is milder than garlic and much more palatable to some people than garlic when used raw as in salads, therefore, it is expected to be useful for a wide range of purposes including healthcare (2, 3).

Numerous studies indicate that garlic (*A. sativum* L), contains antimicrobial agents named allicin containing diallylsulphide and thiosulfinate that are highly effective against major foodborne pathogens (4, 5). Allicin is readily membrane-permeable and undergoes thiol-disulphide exchange reactions with free thiol groups in proteins. It is thought that these properties are the basis of its antimicrobial action (6). It was found that elephant garlic extracts, like other *Allium* species, had eight different thiosulfinates (7), so elephant garlic might be as effective as garlic in terms of antibiotic activity (8, 9), since that it is a variant species of *Allium*. Thus, it might be reasonable to systematically compare the effectiveness of elephant garlic with antibiotics against common bacteria.

It has been proved that garlic can slow down the growth of cancer cells, leading to cell cycle arrest, apoptosis and angiogenesis inhabitation (10). Garlic has been used throughout the stages of treatment in cancer for a long time and it is therefore known as a cancer fighting herb. But there are few reports on the anti-cancer properties and mechanism of elephant garlic.

In this study, MTT assay and cell counting experiments were run to test osteosarcoma cells (U2OS) proliferation and cell activity under exposure of elephant garlic. Flow cytometry assay was conducted on human osteosarcoma cells exposed to elephant garlic extract. By the means of confocal laser scanning microscope, the cytoskeleton of osteosarcoma cells, U2OS, was observed.

## Materials and Methods

***Agar diffusion method assay***

The bacteria namely *Bacillus subtilis, Bacillus thuringiensis, Actinoplanes violaceus, Actinoalloteichus cyanogriseus, Actinomyces aureus, Staphylococcus aureus* and *Escherichia coli*, were supplied by the microbiology lab in Beijing Forestry University. They were reconstituted and their viability and purity were confirmed in agar.

Antibacterial activity of elephant garlic extract was determined by disc diffusion method on nutrient agar medium. Sterile Whatman filter discs (6 mm diameter) were made in nutrient agar plate and inoculums, containing 200 μl of bacteria were spread on the solid plates with a sterile swab moistened with the bacterial suspension. Then, 20 μl each of elephant garlic extract were placed in the discs made in inoculated plates. The treatments also included 20 μl of saline served as the negative control and ampicillin as the positive control. The plates were incubated for 48 hr following standard protocols and zone of inhibition, if any, around the wells were measured in mm (millimeter). Each experiment was repeated for three times (11, 12).

***Extraction*** ***containing fractions from elephant garlic***

To prepare garlic juice, garlic bulbs were separated, peeled and washed with distilled water. After drying in a shed, the clean garlic bulbs were crushed with an electric grinder and the extract was decanted carefully through muslin cloths (13).

***Osteosarcoma cell line and cell culture***

The human osteosarcoma cancer cell line, U2OS, was obtained from the institute of basic medical science, Chinese academy of medical science, androutinely maintained in RPMI-1640 medium supplemented with 10% newborn calf serum and penicillin (100 units/ml)-streptomycin (100 units/ml) at 37°C in a humidified 5% CO_2_ atmosphere (14).

***FACS analysis***

U2OS cells were treated with 0.1% elephant garlic extraction for 24 hr. Then, together with untreated control cells, they were harvested in PBS and 100 μl of the solutions (1 x 10^5^ cells) were taken and stained with the Annexin V-FITC using Apoptosis Detection kit I (BD Biosciences, CA) according to the manufacturer's instructions.

***Cell proliferation assay***

Cell viability was determined by the 3-[4,5- dimethylthiazole-2-yl]-2,5-Diphenyltetra-zolium bromide MTT assay. MTT assays for cell viability were performed in flat-bottomed 96-well plates. After treatment, 20 μl MTT (5 mg/ml) was added to each well. Following further routine incubation for 4 hr, the supernatants were discarded, and 100 μl DMSO was added to each well to dissolve the purple formazan crystals. Subsequently, the optical density (OD) was measured at 570 nm using a Varioskan Flash Multimode Reader (Thermo Fisher Scientific, Waltham, MA, USA). The cell viability was presented as the inhibition rate (IR) that was calculated with the following formula: IR (%) = (1 − OD treatment groups or control groups/OD vehicle control group) × 100%. Cell counting for cell viability was performed in flat-bottomed 96-well plates, after 24 hr of treatment (15).

***Confocal laser scanning microscopy***

Cells were fixed using formaldehyde for 30 min and washed with 0.1% Triton X-100 in PBS for 10 min. FITC-conjugated phalloidin and 4,6-diamidino-2-phenylindole (DAIPI) were used to label F-actin and cell nuclei for 30 min in the dark, respectively. After that, laser scanning confocal microscopy was used to take the images, which were subsequently analyzed with the instrument's software.

***Transwell migration assays***

In this method, 100 μl of the diluted matrigel (BD) was put into upper chamber of 24-well transwell and incubated at 37°C for 6 hr. U2OS cells were treated with 10 µM for 24 hr, cell motility was assessed by the transwell migration assay. A number of 1×10^4^ cells in 100 μl medium was plated in 3–4 replicates in either invasion or control chambers under a chemotactic gradient of serum, overnight. The transwell was removed and fixed using formaldehyde, then stained with DAIPI solution. Cell nuclei on the filters were visualized under a fluorescent microscopy.

***Statistical analysis***

Statistical significance was assessed by two-tailed Student’s *t*-test and reported for a *p* <0.05. Data are expressed as means ± SE.

## Results

***Elephant garlic effects on bacteria proliferation***

The zones of inhibition could be seen against all of the microorganisms tested after 24 hr of growth (Table 1).

The data in Table 1 indicates that there were four kinds of bacteria sensitive to elephant garlic, whose diameter of inhibition zone was more than 21mm.

The study found out that the antibacterial capacity of elephant garlic was not weaker than that of garlic, and it was even stronger on some kind of bacteria (purple actinomycetes, gray actinomycetes). Its antibacterial activity was stronger than ampicillin when applied to *E. coli, B. subtilis, B. thuringiensis, S. actinomycetes*, and *gray actinomycetes* (Table 2).

**Figure 1:**
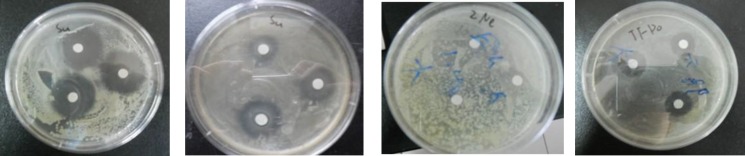
Antibacterial activity of *Allium ampeloprasum L* on *Bacillus thuringiensis* during the course of different treatment

The antibacterial effect is different on different bacteria, and shows dose dependency. Even at a very low concentration (12.5%), elephant garlic still had antibacterial effect on common pathogens bacteria *E. coli* and *S. aureus* (Figure 1).

**Table 1:** Inhibition zones of elephant garlic

	Inhibition zone diametera (mm)			
Bacteria	Garlic	Elephant garlic	Saline	Ampicillin
*E.coli*	23.3±0.2b	18.7±0.2c	6	15.7±0.1c
*S. aureus*	25±0.2b	19.3±0.2c	6	25.7±0.2b
*B. subtilis*	25±0.2b	15.3±0.1c	6	14.7±0.1c
*B. thuringiensis*	31.3±0.3a	21±0.2c	6	20±0.2b
*Actinopla* *. violaceus*	24.3±0.1b	26±0.3b	6	29.3±0.3a
*Actinomy* *. aureus*	24±0.1b	25±0.3b	6	19±0.1c
*Actinoall* *. cyanogriseus*	21.3±0.2c	25.7±0.2b	6	24±0.2b

Values are expressed as mean±SEM of triplicate experiments. Means in the same row with different letters are significantly different (*P < *0*.*05, Tukey’s test

**Table 2:** Inhibitory effect of different concentrations of elephant garlic on bacteria

	Dilution times			
Bacteria	1/1	1/2	1/4	1/8
*E. coli*	+	+	+	+
*S. aureus*	+	+	+	+
*B. subtilis*	+	+	+	-
*B. thuringiensis*	+	+	+	-
*Actinopla* *. violaceus*	+	+	+	-
*Actinomy* *. aureus*	+	+	+	-
*Actinoall* *. cyanogriseus*	+	+	+	-
+Effective, there were fewer than five colonies on the agar plate.				
-Ineffective, there were more than five colonies on the agar plate.				

***The effect of elephant garlic on U2OS cell viability, proliferation and morphology***

**Figure 2:**
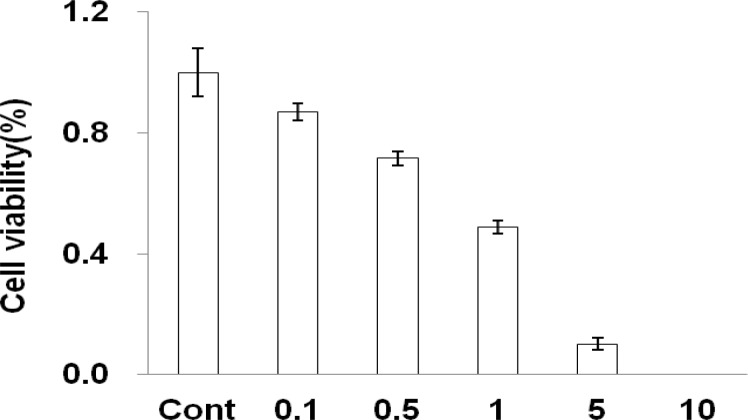
Effect of *A. ampeloprasum *L on viability of U2OS cells (*P*< 0.001

The potential inhibitory effect of the present regimen on the viability of U2OS was evaluated by MTT assays (Figure 2).

The x axis shows the percentage of elephant garlic concentration (% in media). Data are presented as mean±SD. Each in triplicate.

The data demonstrated that elephant garlic depressed U2OS cell viability, dose-dependently. Even at a very low dose (0.1%), the effect was visible (IR = 13%). When the doses were 10%, the viability was decreased to 0% as compared to control.

**Figure 3:**
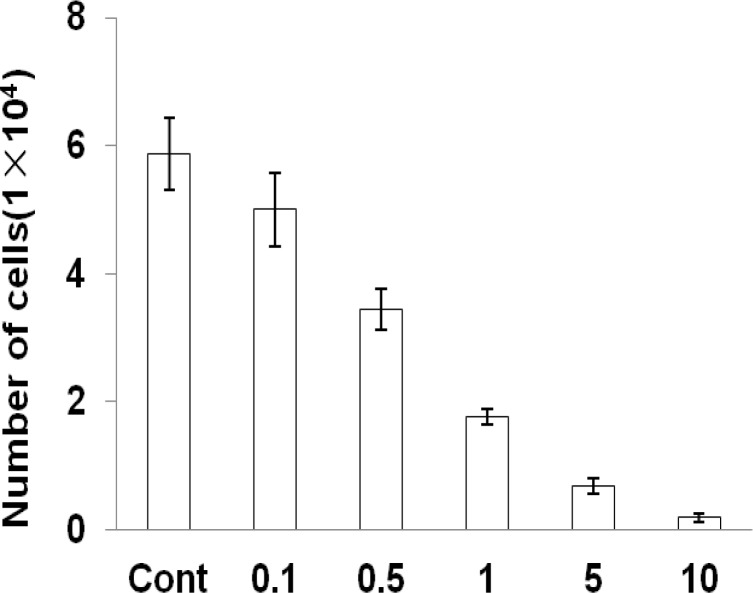
Effect of *A. ampeloprasum *L on proliferation of U2OS cells (*P*< 0.001

By the means of cell counting assay, it was found that elephant garlic preferentially depressed U2OS cell viability. The result suggested that elephant garlic dose-dependently depressed U2OS cell proliferation. Moreover, the bright field image demonstrated that elephant garlic morphology dose-dependently changed (Figure 3).

The x axis shows the percentage of elephant garlic concentration (% in media). Data are presented as mean±SD. Each in triplicate.

**Apoptosis and necrosis induction by elephant garlic in U2OS**

**Figure 4:**
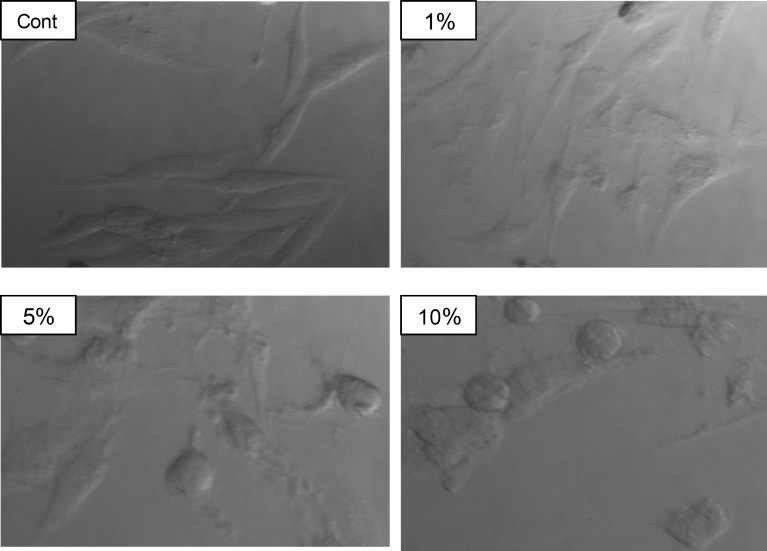
Effect of *Allium ampeloprasum *L on the morphology of U2OS cells

Subsequently, the type of cell death for U2OS cells was identified by FCM assays using FITC-annexin V and PI (propidium iodide) stains. The data demonstrated that elephant garlic (0.1%, for 24 hr) rapidly and substantially increased the early apoptosis rates (8.1% compared to 3.1%) and necrosis rates, and late apoptosis of U2OS cells (20.6% compared to 3.4%) (Figure 4). These results indicated that elephant garlic was inclined to induce apoptosis and necrosis in U2OS cells.

**Figure 5:**
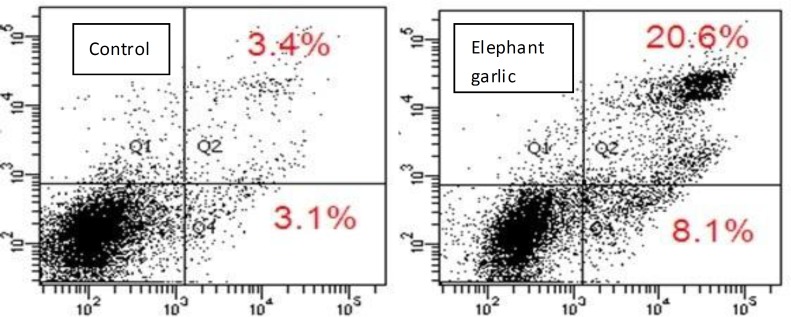
The effect of elephant garlic on apoptosis and necrosis induction in U2OS. High numbers of apoptosis cells are present in the treatment group. The cells were collected after 24 hr of exposure, then stained with FITC and PI (n=4), followed by two-color FACS analysis for PI and FITC. *P* <0.01

**Figure 6:**
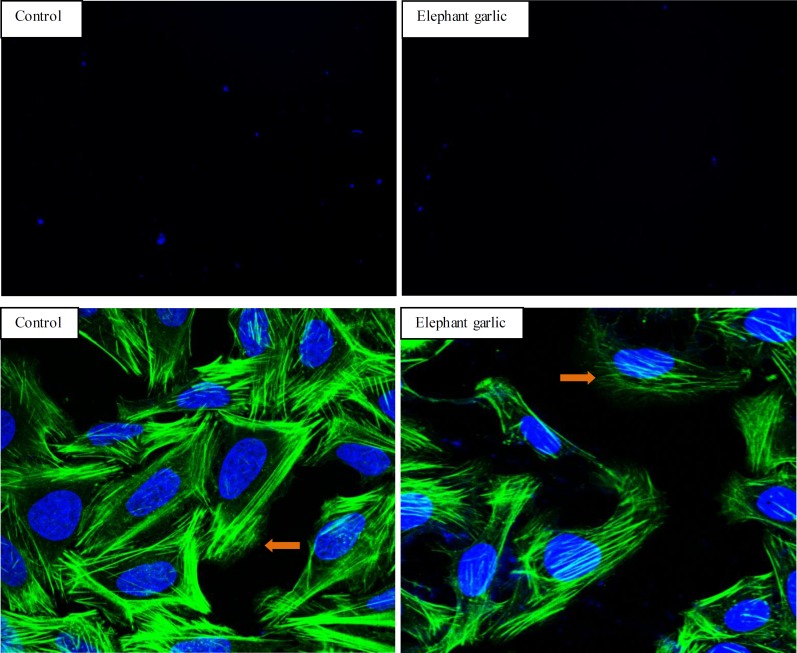
Elephant garlic affects the metastasis of U2OS

***Elephant garlic affects the metastasis of U2OS***

A transwell migration assay revealed that U2OS metastasis was strongly attenuated by elephant garlic (n=3, *P*<0.05) compared to control (n=9, *P*<0.05, Figure 6a). As metastasis is responsible for the major death of osteosarcoma(16), elephant garlic becomes a promising drug in reducing death rate of osteosarcoma.

As detach and become motile is the first step in metastasis (17), and cytoskeleton plays an important role in metastasis process (18). We further ran a confocal assay to explore the mechanism of the reduced metastasis. In the confocal assay, the cytoskeleton of control had clear boundary, and cytoskeleton showed a distinct and clear image suggesting that cells in a well state. Upon treatment with elephant garlic, cells showed a contraction-like figure, and their cytoskeletons were significantly reduced, and mostly fracture-like appearance (Figure 6b). This result suggested that elephant garlic could significantly disassemble the actin fibers and suppress stress fiber formation. In fact, recent study also found that the effects of allicin (diallyl thiosulfinate) on cell polarization, migration, and mitosis are similar to the effects of microtubule-depolymerizing drugs such as nocodazole (19).

Several experiments were recently conducted to investigate the influence of elephant garlic on osteosarcoma cell line, U2OS. We found that elephant garlic affected the proliferation, metastasis, and cytoskeleton of U2OS.

The novelty of this study is that not only the antimicrobial effect of elephant garlic and garlic were examined and compared, but also it was shown that antimicrobial activity of elephant garlic was much stronger than penicillin on some strains. For the first time, it is being shown that the elephant garlic affects proliferation, metastasis, and apoptosis of U2OS, and might produce an impact on the generation of cytoskeleton.

## Discusion

Garlic is a historical medicinal plant (20), which is usually used as diet herb against foodborne pathogens (21, 22) and cancer (23, 24). The use of elephant garlic, a closely related species of garlic, has been barely investigated. In the present study, we investigated the antibacterial activity of elephant garlic and compared it to garlic. This research demonstrated its potential effect on osteosarcoma cells, U2OS. It was confirmed that elephant garlic inhibited the viability of U2OS cells by inducing apoptosis and necrosis, and showed a dose-dependent cytotoxicity. Based on the known mechanisms which mediate the pharmacological effects and physiological activities of garlic, we proposed that the same mechanisms may exert the similar function of elephant garlic.

Firstly, we confirmed that elephant garlic suppresses propagation of daily life-related bacteria. Its antimicrobial activity is not only stronger than garlic, but also stronger than penicillin for some strains. Elephant garlic intake can be of large amount, we propose that it can play a more efficacious role in diet therapy.

Subsequently, we confirmed the anticancer activities of elephant garlic against human osteosarcoma cells, U2OS. We found that elephant garlic depressed U2OS cell viability, proliferation and affected their morphology, and we verified that the inhibitory effect was through the induction of apoptosis and necrosis.

We also found that elephant garlic not only inhibited cancer cells directly via anti-proliferation, but also affected the cancer cells metastasis process to anticancer indirectly. The metastasis was reduced for 66.7% following exposure to elephant garlic, based on detaching and becoming motile is the first step in metastasis. Besides, confocal assay was firstly taken to confirm that the anti-metastasis effect might be through inhibition of the generation of the cytoskeleton. Since rho-associated protein kinase (ROCK) is a key regulator in regulating the shape and movement of cells by acting on the cytoskeleton (25), our data may support a positive regulation of elephant garlic on ROCK and its downstream targets. To reveal the exact interaction of elephant garlic with ROCK further investigations should be done.

## Conclusion

Elephant garlic has potential anti bacterial activity against some common bacteria. Furthermore, evidence shows that the potential use of elephant garlic as an anticancer agent. Further investigation is also required to explore the anticancer mechanism of elephant garlic.
